# Academie de Medicine et des Sciences, Paris

**Published:** 1879-06

**Authors:** 


### ACADEMIE DE MEDICINE ET DES SCIENCES, PARIS.

AT A RECENT MEETING OF THIS BODY, PROFESSOR FIZEAU IN THE CHAIR, AN IMPORTANT COMMUNICATION WAS PRESENTED BY M. PAUL BERT,

*On the possibility of producing, by means of Protoxide of Nitrogen, prolonged insensibility, and on the innocuous qualities of this Anæsthetic.*

This gas, discovered by Sir Humphrey Davy towards the end of the last century, is, at the present day, employed by many practitioners for the purpose of producing insensibility during dental operations. This insensibility can not, however, be allowed to exist for any considerable length of time, for reason: that the moment at which the desired degree of insensibility is gained, symptoms of asphyxia appear, which would soon lead to fatal results. For this reason, the American surgeons have not succeeded in performing lengthened operations on patients under the influence of protoxide of nitrogen, except by administering this gas in such quantities as to produce short, but repeated periods of insensibility, allowing the patient to recover consciousness between each period. This difficulty results from the fact, that insensibility can only be produced by causing the patient to inhale pure protoxide of nitrogen, without any mixture of air; it follows, therefore, that asphyxia and insensibility are produced at one and the same time. M Paul Bert proposes to produce prolonged insensibility, obviating, at the same time, all danger of supervention of asphyxia. The fact, that protoxide of nitrogen must be administered in a pure state, signifies that the tension of this gas, must, in order, that it may penetrate into the organism in sufficient quantity, be subjected to a pressure equal to that of an atmosphere. Under the normal pressure, it is necessary, in order to obtain this gas, that it should be in the proportion of one hundred per cent. If we suppose, however, the patient to be placed in an apparatus in which the pressure is increased to that of two atmospheres, he may be subjected to the required tension by causing him to inhale a mixture of fifty per cent. of protoxide of nitrogen, and fifty per cent. of air; in this manner insensibility would be produced, while the normal amount of oxygen would be maintained in the blood and consequently the normal condition of respiration preserved. "This, says M. Bert, "is what actually occurs." As yet, however he has experimented on animals only. The following is his description of his manner of conducting this experiment: "I enter the cylinder, and there, with an augmentation of pressure equal to that of one and one-fifteenth of an atmosphere, I cause a dog to inhale a mixture of five-sixths of protoxide of nitrogen, and one-sixth of oxygen, a mixture in which, as will at once be perceived, the tension of the so-called laughing gas is exactly equal to that of an atmosphere. Under these conditions, in from two to three minutes, a very short period of agitation, the animal enters into a state of total insensibility; the cornea and conjunctiva may be touched without causing any movement of the eye, of which the pupil is dilated; a sensitive nerve may be laid bare and pinched, or a limb amputated, without causing the slightest movement; the muscular relaxation is extraordinary, and were it not for respiratory movements, which continue with perfect regularity, death would appear to have ensued. This condition may continue for half an hour, or an hour, without any change taking place. During this time the blood retains its red color and its richness in oxygen, the heart its force in rhythmic action, and the temperature its normal state. Any excitation brought to bear on a centripetal nerve induces in the circulation and respiration all those phenomena of reflex action which would occur in the case of an animal in its normal state. In short all the phenomena of the so-called *vegetable life* remain intact, while those of the animal life are for a time abolished; when, at the end of any given period of time, the vessel containing the gaseous mixture is taken away, the animal, at the third or fourth respiration recovers almost immediately sensibility, volition and intelligence, as is sometimes unpleasantly proved by the desire to bite the operator, frequently exhibited by it. When unbound, it moves freely, and rapidly recovers its original gaiety and vivacity. This rapid return to the normal condition, so different from the after-effects when chloroform has been employed, arises from the fact that protoxide of nitrogen does not, like chloroform, enter into chemical combination with the organization, but is simply dissolved in the blood. As soon as the air inhaled ceases to contain this gas it escapes rapidly through the lungs; this I have discovered by analyzing the gases of the blood. The innocuous action of protoxide of nitrogen is clearly proved by these experiments. On the one hand, the anæsthesia affects only the medullary sensibility, leaving intact the reflex movements of organic life, the suppression of which, easily resulting through the employment of chloroform, can alone lead to fatal results. The harmlessness of this gas is still more decisively proved by the comparatively few accidents which have occurred from its administration in numerous cases by the dentists (the cases may be counted by hundreds of thousands), and the more so, that this gas has frequently been administered without prudence and by incompetent persons, under conditions in which the intervention of asphyxia would augment the dangers, if such exist, of anæsthesia. I feel myself, therefore, fully authorized through my experiments on animals, in recommending to surgeons in the most emphatic manner, the employment of protoxide of nitrogen under pressure, when insensibility of long duration is required. I assure them that by measuring, according to the method already indicated by me, the barometrical pressure, and the centesimal composition of the mixture in such a manner as to produce the pressure of an atmosphere for the protoxide of nitrogen, and the normal pressure of the air for the oxygen, they will obtain as complete insensibility and muscular relaxation as they can possibly desire, with rapid recovery of sensibility, and consequently the perfect consecutive well being of the patient. The method of administration is singularly convenient, as, in case of any of those slight inequalities resulting from special degrees of susceptibility between different individuals, which invariably occur, it is sufficient either to diminish or increase the barometrical pressure, and this may with ease be effected by means of a cork. I see but one serious difficulty, that of obtaining the necessary apparatus, which I must admit to be an insuperable one to army surgeons attached to regiments on active service. As the greater number of important operations take place in large towns, however, and as these are generally supplied with baths in which compressed air is employed, I confess I can see no great difficulty in providing in them a room large enough to contain the patient, the operator, and some dozen assistants, while the expense, some twelve thousand francs, would not form a very formidable item in the accounts of any hospital. These difficulties however, are merely questions of secondary importance, the solution of which concerns the surgeons who, in the future, will employ this anæsthetic; on them also will fall the burden of arranging those numerous questions of detail, which ever arise on the introduction of any new therapeautical agent. I have fulfilled my duty as a physiologist in pointing out this agent, in demonstrating the immense advantages which would accrue from its employment, and in insisting on its innocuous qualities, which can be explained with such wonderful facility."

